# Symbolic Numerical Magnitude Processing Is as Important to Arithmetic as Phonological Awareness Is to Reading

**DOI:** 10.1371/journal.pone.0151045

**Published:** 2016-03-04

**Authors:** Kiran Vanbinst, Daniel Ansari, Pol Ghesquière, Bert De Smedt

**Affiliations:** 1 Parenting and Special Education Research Unit, Faculty of Psychology and Educational Sciences, University of Leuven, Leuven, Belgium; 2 Numerical Cognition Laboratory, Department of Psychology & Brain and Mind Institute, University of Western Ontario, London (ON), Canada; University of Melbourne, AUSTRALIA

## Abstract

In this article, we tested, using a 1-year longitudinal design, whether symbolic numerical magnitude processing or children’s numerical representation of Arabic digits, is as important to arithmetic as phonological awareness is to reading. Children completed measures of symbolic comparison, phonological awareness, arithmetic, reading at the start of third grade and the latter two were retested at the start of fourth grade. Cross-sectional and longitudinal correlations indicated that symbolic comparison was a powerful domain-specific predictor of arithmetic and that phonological awareness was a unique predictor of reading. Crucially, the strength of these independent associations was not significantly different. This indicates that symbolic numerical magnitude processing is as important to arithmetic development as phonological awareness is to reading and suggests that symbolic numerical magnitude processing is a good candidate for screening children at risk for developing mathematical difficulties.

## Introduction

Reading and arithmetic represent the core subjects of the educational curriculum in primary schools. A great amount of research has been devoted to the predictors of reading ability and this research has revealed that phonological awareness, or the conscious sensitivity to the sound structure of language, underlies individual differences in learning to read [1], [2]. Considerably less research has been done on the prediction of arithmetic ability, but over the past decade there has been an increasing interest in this question [3]. In 1999, Gersten and Chard [4], theoretically suggested, that numerical magnitude processing skills, or people’s elementary intuitions about quantity and their ability to understand the meaning of Arabic digits, might be “*an analog as important to mathematics learning as phonemic awareness has been to the reading research field”* (p. 18). Since then, numerous studies have shown cross-sectional and longitudinal associations between numerical magnitude processing and arithmetic ability [5], [6], [7], [8], [9], [10], [11], but it has never been tested whether the strength of this association is similar to the well-established phonological awareness-reading association, as suggested by Gersten and Chard [4]. This was precisely the goal of the present study.

Increasing evidence stresses the role of numerical magnitude processing for children’s growth in mathematics, but the respective roles of symbolic versus nonsymbolic numerical magnitude processing remain a source of debate. For example, Chen and Li [12] as well as Fazio, Bailey, Thompson and Siegler [13] focused in their meta-analyses on the association between nonsymbolic numerical magnitude processing skills and mathematical competence, showing moderate associations between both concepts. De Smedt et al. [3] suggested in their narrative review on the association between nonsymbolic and symbolic numerical magnitude processing abilities and mathematical competence, that these associations were more robust for symbolic than for nonsymbolic measures. Testing this assumption statistically, Schneider et al. [14] ran a meta-analysis that included both nonsymbolic and symbolic measures of numerical magnitude processing and revealed that the effect size of the association between symbolic numerical magnitude processing (*r* = .302, 95% CI: .243 - .361) and mathematical competence was significantly larger compared to the effect size of the association with nonsymbolic numerical magnitude processing (*r* = .241, 95% CI: .198 - .284). Against this background, the present study focused on symbolic numerical magnitude processing as a major determinant of children’s concurrent and future competence in mathematics.

De Smedt and colleagues [3] also suggested in their narrative review, that (symbolic) numerical magnitude processing might impact more on some specific mathematical subdomains compared to others. Schneider et al. [14] formally tested this suggestion in their meta-analysis by investigating to what extent the measure of mathematical competence moderated the association between symbolic numerical magnitude processing and mathematical competence. Their analyses revealed that the strength of the association differed according to the mathematical subdomain under investigation and, importantly, that this strength was particularly strong for elementary mathematical skills, such as arithmetic. Arithmetic comprises a major building block for subsequent growth in mathematics [15] and arithmetic deficits constitute the hallmark of children with mathematical learning difficulties or dyscalculia [16]. The present study, therefore, focused on arithmetic as mathematical subdomain.

In view of the above, the goal of this study was to empirically test whether symbolic numerical magnitude processing contributes to arithmetic (development) like phonological awareness contributes to reading, as was suggested by Gersten and Chard [4]. We restricted our focus to arithmetic rather than mathematical achievement more broadly construed, which is also similar to studies in reading where the impact of phonological awareness has been mainly investigated in the context of children’s proficiency to decode words [2],which is one specific and crucial subdomain of reading development that underlies other aspects of reading as for example, reading comprehension.

We used a longitudinal design, testing children at the start of third (Time 1) and fourth (Time 2) grade. We evaluated their symbolic numerical magnitude processing skills with a standard symbolic comparison task in which children have to indicate as quickly as possible the larger of two Arabic digits [6], [17]. Likewise, we used a classic phoneme deletion task to investigate children’s phonological awareness [18], a measure that has been widely used in reading research [1], [2]. At both time points we measured children’s arithmetic and reading abilities.

Our first aim was to test the expected cross-sectional and longitudinal associations between symbolic comparison and arithmetic as well as between phonological awareness and reading, and to verify whether these associations were unique. Our second aim was to compare the strength of the symbolic numerical magnitude processing-arithmetic versus the phonological awareness-reading associations. This was done with classic regression analyses as well as Bayesian hypothesis testing [19].

## Method

### Participants

Participants came from an ongoing longitudinal study, in which three schools participated. The initial sample comprised 74 children (29 boys, 45 girls; *M*_age_ = 8 years and 2 months, *SD =* 2 months) at Time 1 (2011). One year later, at Time 2 (2012) in fourth grade, data were available for 67 children. Missing data were due to illness or changing schools. Data of all 74 participants at Time 1 were used in the cross-sectional analyses. In the subsequent longitudinal analyses only those participants whose data were available at Time 1 and Time 2 were included. The 7 participants with missing data at Time 2 had lower reading ability and performed more poorly in single-digit arithmetic at Time 1, although in view of the small sample of missing participants (*n* = 7), such comparisons should be treated with great caution. However, the absence of these participants at the lower end of the continuum of scholastic abilities might explain why longitudinal associations were less strong compared to cross-sectional associations (due to a decrease in subject-variability). All children had Dutch, a language with a relatively transparent orthography, as their mother tongue. They came predominantly from middle- to upper middle-class families and their intellectual ability, as determined by Raven’s matrices [20], was within the normal range (*M* = 107.97, *SD =* 11.65). None of the participants repeated a grade.

### Ethics statement

Parents of all children received an information sheet on the study and provided written informed consent for their child. Given the age of our participants, children did not sign written consent but they all gave verbal agreement before undertaking the different experiments and tasks. The study and consent procedures were approved by the ethics committee of the KU Leuven (University of Leuven).

### Materials

Materials were computer tasks designed with the E-prime 1.0 [21], paper-and-pencil tasks and standardized tests.

#### Symbolic numerical magnitude processing

Symbolic numerical magnitude processing skills were measured with a classic comparison task. In this task, children had to compare two simultaneously presented Arabic digits, displayed on either side of a 15-inch computer screen. They had to indicate the larger one by pressing a key on the side of the larger digit. Stimuli comprised all combinations of numbers 1 to 9, yielding 72 trials. The position of the largest digit was counterbalanced. Each trial was initiated by the experimenter and started with a central 200ms fixation point, followed by a blank of 800ms. Stimuli appeared 1000ms after trial initiation and, and remained visible until response. Response times and answers were registered. To familiarize children with the key assignments, three practice trials were presented.

#### Phonological awareness

A classic phoneme deletion task, that has previously been used in reading research in Dutch [20], [22] as well as other populations of a similar age [23], was administered to assess children’s phonological awareness. Children were presented with 28 one-syllable Dutch-like nonwords (an adaptation of de Jong & van der Leij [24] and were asked to delete a particular phoneme of the nonword. For the first 10 items, the deletion of the phoneme resulted in the disclosure of an existing word (e.g., DROOS without /d/). For the next 18 items, the residual phonological string remained meaningless after phoneme deletion (e.g. WAPT without /t/). Each subtest was preceded by two practice items to make the child familiar with task administration. Each correctly solved item was rewarded with one point (maximum = 28).

#### Arithmetic

Children’s arithmetic was assessed with an experimental single-digit addition and subtraction task. The horizontally presented stimuli were selected from a standard set of single-digit arithmetic problems [25], which excludes tie problems (e.g., 6 + 6) and problems containing 0 or 1 as operand or answer. For addition, one of each pair of commutative problems was selected, resulting in a set of additions in which the position of the largest operand was counterbalanced. Subtraction problems were constructed from the complements of the additions problems. Children had to perform both accurately and quickly on a set of 28 problems per operation. Responses were verbal. A voice key registered the child’s response time per trial, after which the experimenter recorded the answer. Two practice trials were presented to familiarize children with task administration.

At Time 2, we additionally evaluated children’s competence in solving more complex arithmetic, by assessing the Tempo Test Arithmetic (TTA) [26], a normed standardized achievement test of arithmetic [27] comparable to the Arithmetic Fluency test of the Woodcock Johnson [28]. The addition subtest as well as the subtraction subtest of the TTA was presented. Each subtest involved 40 problems of increasing difficulty: Children had to solve as many single-digit and multi-digit additions or subtractions as possible within one minute. The score on this test is the number of correctly solved problems on each subtest within the time-limit per subtest (maximum = 80). This test combines speed and accuracy into one index score. Performance on this standardized test at Time 2 correlated highly with performance on the experimental single-digit arithmetic task at the same measurement point (Time 2) as well as the measurement point one year earlier (Time 1). These results can be considered as a validation of the previously described experimental single-digit addition and subtraction tasks.

#### Reading ability

Reading ability was assessed by the normed and standardized Dutch One-Minute-Test version B [29], which is widely used in Flanders. Children had to read a list of 116 one- up to five-syllable single words of increasing difficulty as correctly and quickly as possible. The total score was the number of words read correctly within one minute. Similar to the TTA, this test combines speed and accuracy into one index score.

#### Intellectual ability

Raven’s Standard Progressive Matrices [20] was administered as a measure of intellectual ability. For each child, a standardized score (*M =* 100, *SD* = 15) was calculated.

### Procedure

All tasks were individually administered in a quiet room at the children’s own school, except for the Raven’s matrices and the TTA, which were group-based. Task order was fixed for all participants. At both Time 1 and 2, the single-digit arithmetic task and reading test were administered. The symbolic comparison task and the phoneme deletion task were only administered at time 1. The TTA was only assessed at time 2. Intellectual ability was assessed in third grade.

## Results

Trials with incorrect responses or incorrect voice-key registrations (< 4% of all trials) were excluded from response time analyses. For the symbolic comparison task, trials for which children had a response time lower than 300ms or higher than 5000ms were discarded (< 1% of all trials). For single-digit arithmetic, trials deviating more than 3 *SD*s from the average participant’s response time and trials with a response time below 500ms (< 0.5% of all trials) were excluded.

### Descriptive Analyses

Descriptive statistics and reliabilities of the measures collected at both time points are presented in Table 1. Repeated measures ANOVA with Time (Time 1 vs. Time 2) as within-subject factor were conducted on children’s performance on the single-digit arithmetic task, indicating that accuracy, *F*(1, 66) = 44.72, *p* < .001, *η*^*2*^_*p*_ = .40, and response time, *F*(1, 66) = 118.57, *p* < .001, *η*^*2*^_*p*_ = .64, improved over time. The same was true for their reading ability, *F*(1, 66) = 316.06, *p* < .001, *η*^*2*^_*p*_ = .83.

**Table 1:** Descriptive statistics and reliabilities of the measures collected at Time 1 (*n =* 74) and Time 2 (*n =* 67).

	*M*	*SD*	Maximum Possible	Reliability
**Time 1**				
Symbolic comparison				
Accuracy (proportion correct)	0.94	0.04	1.00	.76^a^
Response time (ms)	947.84	212.19		.94^a^
Phonological awareness	18.91	6.09	28.00	.92^b^
Single-digit arithmetic				
Accuracy (proportion correct)	0.91	0.07	1.00	.65^a^
Response time (ms)	3935.62	1641.31		.91^a^
Reading	47.89	12.42	116.00	.90^c^
**Time 2**				
Single-digit arithmetic				
Accuracy (proportion correct)	0.97	0.03	1.00	
Response time (ms)	2421.99	896.69		
Tempo Test Arithmetic	43.11	7.53	80.00	.92^d^
Reading	60.57	12.16	116.00	.90^c^

^a^ Odd-even reliability in the current sample calculated at Time 1.

^b^ Cronbach alpha [18].

^c^ Derived from test manual [30].

^d^ Derived from test manual [31].

### Correlational Analyses

Pearson correlation coefficients were calculated to examine the associations between the different measures under study (Table 2; S1 Fig). For the single-digit arithmetic task and the symbolic comparison task, we calculated a score that combined response time and accuracy into one index by dividing an individual’s mean response time by his/her mean accuracy [32]. For example, this combined index allowed us to provide the best possible picture of a child’s arithmetic ability, i.e. an index of children’s fluency or the ability to be fast and accurate. As a result, data from the experimental tasks were similar to the standardized test data, as all measures were speeded and they all combined speed and accuracy into one score. This additionally allowed for a better comparison of the symbolic-arithmetic association and phonology-reading association.

**Table 2:** Associations between all measures under study.

	1	2	3	4	5	6	7	8
1 Symbolic comparison Time 1		-.25*	.49***	-.22	.45***	-.33**	-.18	-.09
2 Phonological awareness Time 1			-.28*	.44***	-.21	.12	.42***	.32**
3 Single-digit arithmetic Time 1				-.32**	.72***	-.62***	-.31*	-.24*
4 Reading Time 1					-.42***	.40**	.90***	.02
5 Single-digit arithmetic Time 2						-.74***	-.43***	-.23
6 TTA Time 2							.43***	.09
7 Reading Time 2								.01
8 Intellectual ability								

Time 1 (*n* = 74); Time 2(*n* = 67); TTA = Tempo Test Arithmetic.

* *p <* .05.

** *p <* .01.

*** *p* < .001.

The data in Table 2 reveal, as expected, significant cross-sectional and longitudinal associations between symbolic comparison and arithmetic, on the one hand, and between phonological awareness and reading on the other hand, and these associations are depicted in S1 Fig. Bayes factors of important correlations are indicated in Table 3 and Table 4 below.

**Table 3:** Regression analyses and Bayes factors explaining cross-sectional variance in arithmetic and reading at Time 1 (*n =* 74).

	Predictors	*β*	*t*	*P*	Unique *R*^*2*^	Bayes Factor
Single-digit	Symbolic comparison	.41	4.00	.000	.16	1456.33
Arithmetic^a^	Phonological awareness	-.02	-0.14	.890	.00	3.24
	Reading	-.22	-1.95	.005	.04	7.92
	Intellectual ability	-.19	-1.83	.071	.03	1.62
Reading^b^	Symbolic comparison	-.02	-0.13	.897	.00	1.19
	Phonological awareness	.43	3.83	.000	.16	294.37
	Single-digit arithmetic	-.24	-1.95	.055	.04	7.92
	Intellectual ability	-.18	-1.62	.109	.03	0.24

^a^ *F*(4, 69) = 8.23, *p <* .001, *R*^*2*^ = .32.

^b^ *F*(4, 69) = 6.34, *p* < .001, *R*^*2*^ = .27.

**Table 4:** Longitudinal regression analyses and Bayes factors predicting children’s arithmetic and reading at time 2 (*n* = 67).

	Predictors	*β*	*t*	*p*	Unique *R*^*2*^	Bayes Factor
Single-digit	Symbolic comparison T1	.37	3.55	.001	.13	156.87
arithmetic T2^a^	Phonological awareness T1	.10	0.86	.393	.01	0.83
	Reading T2	-.41	-3.57	.001	.13	88.68
	Intellectual ability	-.23	-2.06	.044	.04	1.21
TTA T2^b^	Symbolic comparison T1	-.27	-2.40	.019	.07	7.07
	Phonological awareness T1	-.15	-1.17	.248	.02	0.37
	Reading T2	.45	3.69	.000	.16	103.51
	Intellectual ability	.11	0.91	.366	.01	0.31
Reading T2^c^	Symbolic comparison T1	.05	0.47	.642	.00	0.62
	Phonological awareness T1	.42	3.77	.000	.15	58.94
	Single-digit arithmetic T2	-.42	-3.57	.001	.14	88.68
	Intellectual ability	-.23	-2.08	.042	.05	0.25

T1 = Time 1; T2 = Time 2; TTA = Tempo Test Arithmetic.

^a^ *F*(4, 62) = 9.07, *p* < .001, *R*^*2*^ = .37.

^b^ *F*(4, 62) = 5.88, *p* < .001, *R*^*2*^ = .28.

^c^ *F*(4, 62) = 8.13, *p <* .001, *R*^*2*^ = .34.

We additionally compared the strength of these independent correlations obtained from the same sample by means of the Williams-Steiger test [33], [34]. At the cross-sectional level, the strength of the association between symbolic comparison and single-digit arithmetic is not significantly different from that of the association between phonological awareness and reading, *z* = 0.31, *p* = .756. Similarly, the longitudinal association between symbolic comparison and single-digit arithmetic is not significantly different from the longitudinal association between phonological awareness and reading, *z* = 0.22, *p =* .825. Similar results are obtained when the TTA is used instead of the single-digit arithmetic, *z* = 0.55, *p* = .582.

### Additional analyses

Multiple linear regression analyses were performed to assess the amount of unique variance in arithmetic and reading explained by symbolic comparison and phonological awareness, respectively. To this end, all these predictors and intellectual ability were entered simultaneously into each regression. In addition to the null-hypothesis significance testing, we calculated the Bayes factors for each predictor with the BayesFactor package of Morey, Rouder, and Jamil [35] implemented in R, in order to quantify the support that the data provide for the prediction of arithmetic versus reading.

Table 3 displays the results of the regression analyses as well as Bayes factors indicating the unique contribution of each predictor for cross-sectional variance in arithmetic and reading at Time 1 (*n* = 74). Symbolic comparison explains a significant amount of unique variance in children’s single-digit arithmetic. Likewise, phonological awareness explains a significant unique amount of variance in reading. The absolute size of the Bayes factors can be used to compare the evidential strength of the observed associations. To interpret these Bayes factors, we used the recommendations of Andraszewicz et al.[19], which highlight that factors that are larger than 100 indicate extremely strong evidence for a given hypothesis. The Bayes factors provided in Table 3 illustrate extremely strong associations between symbolic comparison and single-digit arithmetic as well as between phonological awareness and reading. A direct comparison of these Bayes factors even indicates that although the evidence is strong for both, symbolic comparison is an at least as strong predictor of single-digit arithmetic than phonological awareness is of reading.

Table 4 displays the regression analyses and Bayes factors illustrating how task performance at Time 1 explains longitudinal variance in arithmetic and reading at Time 2 (*n* = 67). Single-digit arithmetic is uniquely predicted by symbolic comparison one year earlier. Bayes factors show that it is the strongest predictor of single-digit arithmetic (Bayes factor larger than 100). Data on the TTA are similar but less powerful according to the interpretation system of Andraszewicz et al. [19]: Symbolic comparison uniquely predicts TTA, but its prediction is only moderate (Bayes factor between 3 and 10). Reading ability is uniquely predicted by phonological awareness one year earlier. Bayes factors indicate that the association between phonological awareness and reading is very strong (Bayes factor between 30 and 100).

## Discussion

It has been suggested that symbolic numerical magnitude processing is as important to arithmetic as phonological awareness is to reading [4], but this analog has never been tested empirically. This was precisely the goal of this longitudinal study. First, we replicated prior cross-sectional [6], [7], [9], [36], and longitudinal [5], [10], [37], [38] studies showing significant unique associations between symbolic numerical magnitude processing and arithmetic. Our results are in line with the narrative review by De Smedt et al. [3] and the meta-analytic findings of Schneider et al. [14] and convincingly indicate that symbolic numerical magnitude processing is a powerful domain-specific predictor of children’s arithmetic development. Consistent with the far more extensive reading literature, our data confirm that phonological awareness is a significant unique and domain-specific predictor of reading (decoding) ability [1].

Extending the existing body of knowledge, we contrasted the strength of the correlation between symbolic numerical magnitude processing and arithmetic with the well-established phonological awareness-reading association. A direct comparison of these correlation coefficients reveals that they are not significantly different from each other, both cross-sectionally and longitudinally. Bayesian hypothesis testing even indicates that symbolic numerical magnitude processing is at least as important to arithmetic as phonological awareness is to reading. These data support the idea of Gersten and Chard [4].

The predictive value of symbolic numerical magnitude processing was observed for two independent measures of arithmetic, which adds to the generalizability of our findings. Analysis of the Bayes factors shows that symbolic comparison relates more strongly to single-digit arithmetic than to the TTA measure, which contains single- as well as multi-digit arithmetic problems. It is known that children (but also adults) frequently solve single-digit arithmetic with direct fact retrieval, whereas procedural strategies are more often used for solving multi-digit arithmetic. Consequently, our results might reflect the previously demonstrated link between proficient symbolic numerical magnitude processing skills and successful arithmetic fact retrieval [9], [10], [38]. The link between symbolic numerical magnitude processing and multi-digit arithmetic might be less straightforward, depending on the type of procedure that is used to perform the calculation [39].

It must be highlighted that the conclusion of this study only applies to children’s symbolic numerical magnitude processing skills, whereby it remains to be explored whether such strong association with arithmetic would also be found for other numerical capacities, such as subitizing, nonsymbolic numerical magnitude processing, spontaneous focusing on numerosity (SFON), counting procedures or counting principles. Future research should consider symbolic numerical magnitude processing together with additional important numerical capacities, in order to determine the unique contribution of each towards children’s arithmetic development. Moreover, such research would refine the effect of symbolic numerical magnitude processing, given that its association with arithmetic ability might possibly not be as strong if some of these additional numerical capacities were included [13, 40, 41, 42, 43].

A great amount of research has revealed that phonological awareness underlies children’s reading acquisition, but also their growth in arithmetic [23], [44], [45], [46]. Our data confirm that children’s phonological skills play a role in children’s arithmetic development, but suggest, however, that phonological processing skills play a less prominent role in children’s arithmetic development compared to simultaneously considered symbolic numerical magnitude processing skills.

It is important to highlight that the language in which the children were tested, i.e. Dutch, has a relatively transparent orthography. In transparent languages the predictive value of phonological awareness decreases over age [22], [24], [47], compared to languages with a more irregular orthography, such as English [48]. The children in the current study were already in third and fourth grade, which might underestimate the predictive value of phonological awareness compared to less transparent languages. Future studies should therefore replicate the current study in a non-transparent orthography, such as English.

In summary, our longitudinal findings indicate that symbolic numerical magnitude processing is an at least as powerful predictor of arithmetic development as phonological awareness is to reading. This predictor can be measured early in children’s academic career (e.g., start of formal schooling, Vanbinst et al. [10]. Screening measures, to quickly and easily assess symbolic numerical magnitude processing skills in classroom settings, have been developed and validated [49] and such measures might be particularly helpful for identifying children at risk for developing mathematical difficulties.

## Supporting Information

S1 Dataset Dataset study Symbolic numerical magnitude processing is as important to arithmetic as phonological awareness is to reading. (XLSX)

S1 Fig Scatterplots showing significant associations between symbolic comparison and single-digit arithmetic (Panel a), phonological awareness and reading (Panel b), and symbolic comparison and TTA (Panel c). Left-sided graphs represent cross-sectional associations (Time 1) and right-sided graphs represent longitudinal associations (Time 1—Time 2). BF = Bayes Factors.
(PNG)
