# Supplementary figures and images for: Symbolic Numerical Magnitude Processing Is as Important to Arithmetic as Phonological Awareness Is to Reading
Source: PLoS One. 2016 Mar 4;11(3):e0151045. doi: 10.1371/journal.pone.0151045 (PMC4778857; doi:10.1371/journal.pone.0151045)

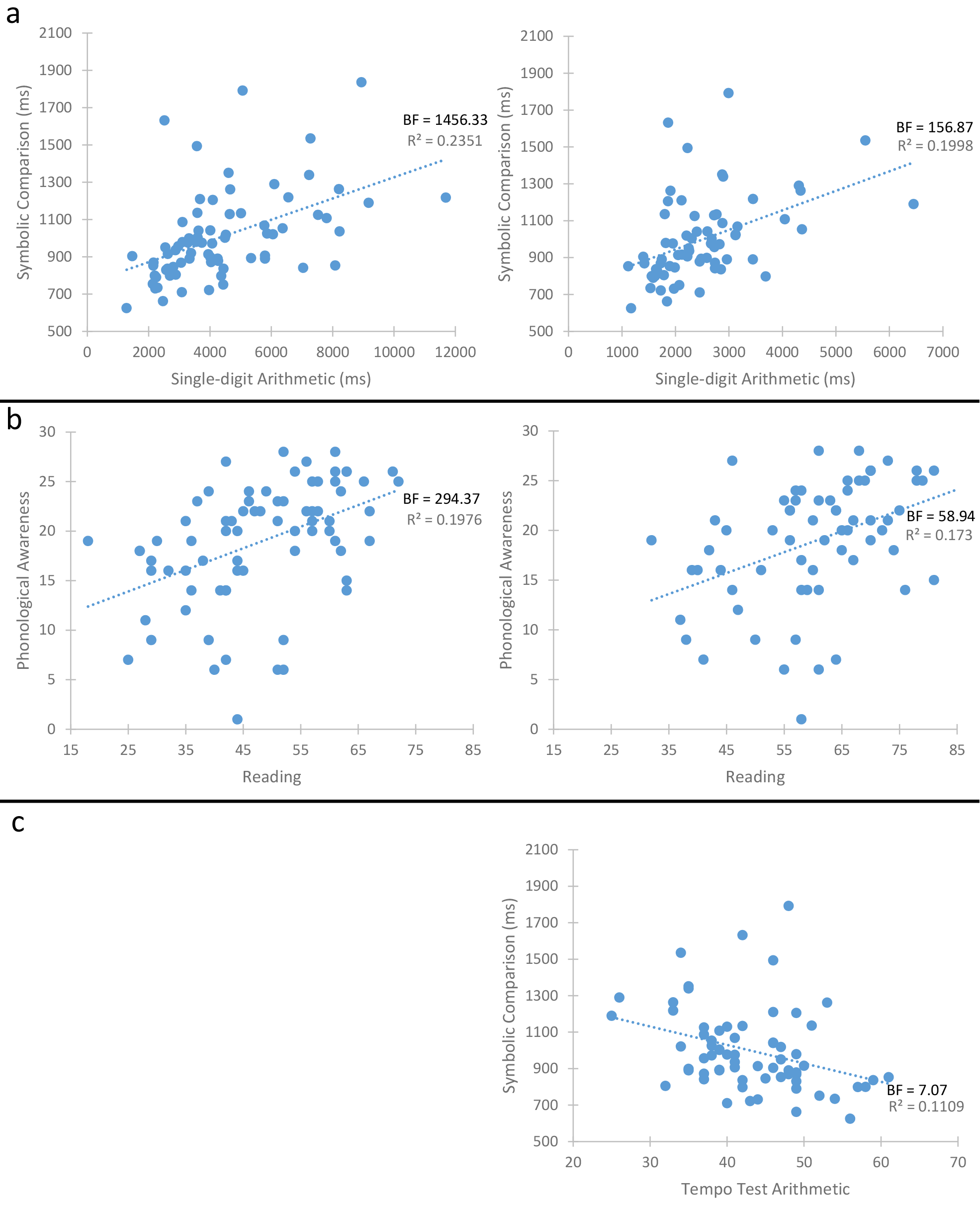

Supplement: S1 Fig — Left-sided graphs represent cross-sectional associations (Time 1) and right-sided graphs represent longitudinal associations (Time 1—Time 2). BF = Bayes Factors. (PNG) [file pone.0151045.s002.png]
